# The complete mitochondrial genome of *Osmanthus fragrans* (Lamiales, Oleaceae) from China

**DOI:** 10.1080/23802359.2021.1942265

**Published:** 2021-06-21

**Authors:** Zhaoxuan Wang, Rengang Zhang

**Affiliations:** a Shijiazhuang People’s Medical College, Shijiazhuang, China; b Shandong Ori-Gene Science and Technology Co., Ltd., Weifang, China

**Keywords:** Lamiales, mitochondrial genome, Oleaceae, *Osmanthus fragrans*, phylogenomics

## Abstract

*Osmanthus fragrans* is a well-known ornamental tree with high medicinal and edible values. In this study, the complete mitochondrial genome sequence of *O. fragrans* was assembled, annotated, and analyzed using phylogenomic methods. The complete mitochondrial genome of *O. fragrans* was 563,202 bp in length and displays an overall GC content of 44.58%. Sixteen chloroplast-derived segments with an average length of 1260 bp were identified. The complete mitochondrial genome contained 74 genes in total, including 44 protein-coding, three rRNA, and 27 tRNA genes, among which seven protein-coding and six tRNA genes were chloroplast-derived. Phylogenetic analysis showed that *O. fragrans* was closely related to *Chionanthus rupicola* within the Oleaceae. This study could provide genomic resources for a better understanding of *O. fragrans* and further studies on the evolution of Oleaceae.

*Osmanthus fragrans* Lour. (sweet osmanthus, Oleaceae) is a well-known evergreen ornamental tree that produces small white, yellow, or orange flowers with a sweet, rich fragrance (Xiang and Liu 2008). It is also edible and has medicinal values (Zhou et al. 2017). In previous phylogenetic studies, *O. fragrans* was clustered with *Chionanthus retusus* based on nuclear ribosomal DNA sequences (Besnard et al. 2009), which is inconsistent with the results based on plastid sequences (Besnard et al. 2009; Duan et al. 2019). In this study, the complete mitochondrial genome sequence of *O. fragrans* was assembled and characterized to reveal the evolutionary history of *O. fragrans* based on a mitochondrial phylogenomic analysis.

The fresh leaves were collected from a tree in the Shijiazhuang People’s Medical College (Hebei, China) (N 37.99°, E 114.45°). The voucher specimen (accession no. PMC160614) was deposited in the Shijiazhuang People’s Medical College (http://www.sjzrmyz.com/, Jie Guo, 876538268@qq.com). Genomic DNA was extracted using a modified CTAB method (Doyle and Doyle 1987). The extracted DNA was fragmented for Illumina library construction by Illumina TruSeq DNA sample prep kit and then sequenced on the HiSeq X Ten platform (Illumina Inc., San Diego, CA, USA). The complete mitochondrial genome sequence of *O. fragrans* was assembled with GetOrganelle v1.6.2e (Jin et al. 2020) and annotated with the OGAP pipeline using the default settings (https://github.com/zhangrengang/OGAP). The draft annotations were then adjusted manually with the assistance of BLAT (Kent 2002).

The complete mitochondrial genome of *O. fragrans* was assembled as a circular molecule 563,202 bp in length (GenBank accession no. MW645067), with an overall GC content of 44.58%. Sixteen chloroplast-derived segments were identified with lengths ranging from 190 to 3349 bp and an average length of 1260 bp. A total of 74 genes were annotated, including 44 protein-coding, three rRNA, and 27 tRNA genes. Among these, seven protein-coding and six tRNA genes were in the above-mentioned chloroplast-derived segments. The mitogenome size and composition of *O. fragrans* were similar to the four related taxa in the Oleaceae (Figure 1), which contained 37–48 protein-coding, three rRNA, and 20–26 tRNA genes with a length ranging from 658,522 (*Hesperelaea palmeri*) to 848,451 bp (*Ligustrum quihoui*) and a GC content of 44.5–44.8% (Van de Paer et al. 2016, 2018).

**Figure 1.**
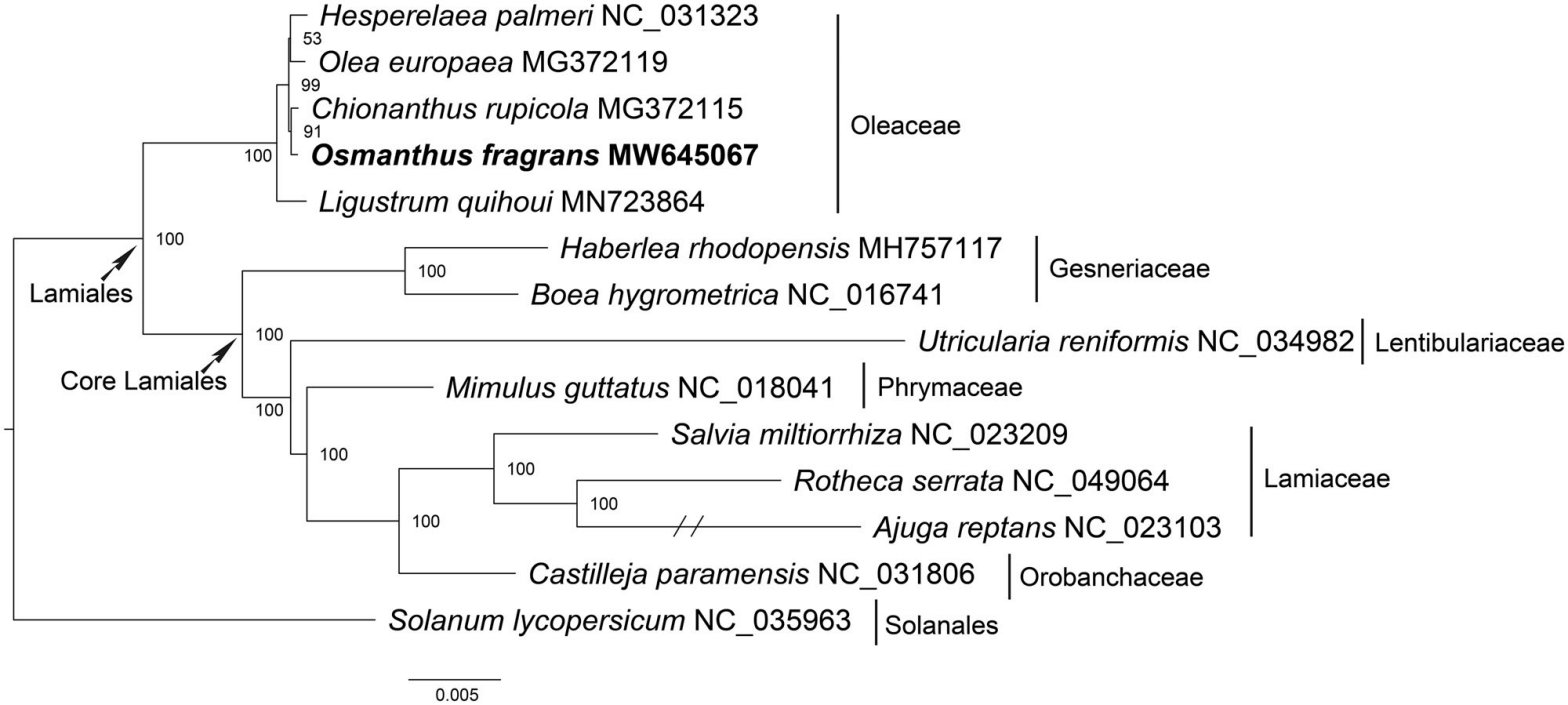
Phylogenetic tree inferred by maximum-likelihood method based on mitochondrial protein-coding gene sequences of *O. fragrans* and other 12 species within the order Lamiales. *Solanum lycopersicum* was served as the outgroup. Numbers in the nodes are the bootstrap values from 1000 replicates. The branch of *Ajuga reptans* was truncated as it was too long.

The phylogenetic analysis was performed with the mitochondrial genomes from 12 other species in the order Lamiales, designating *Solanum lycopersicum* (Solanales) as the outgroup. The 37 mitochondrial protein-coding genes of these species were aligned by using MAFFT v7.471 (Standley and Katoh 2013) and the alignment was trimmed with TrimAl (Capella-Gutierrez et al. 2009). The maximum likelihood phylogenetic tree was reconstructed by using the software IQ-TREE v1.6.5 (Nguyen et al. 2015) based on the best-fit model of TVM + F + R3 and 1000 bootstrap replicates. The phylogenetic tree showed that *O. fragrans* was closely related to *Chionanthus rupicola* and formed a monophyletic clade with four other species classified in the Oleaceae. This monophyletic clade was situated in a basal position of the Lamiales (Figure 1). The phylogenetic relationships within the Lamiales were consistent with previous chloroplast-based studies (Duan et al. 2019; Zhu et al. 2020). In summary, this study extended our knowledge to *O. fragrans* and provided a reference for further research on the phylogeny and evolution of the family Oleaceae as well as the order Lamiales.

## Data Availability

The genome sequence data that support the findings of this study are openly available in GenBank of NCBI at http://www.ncbi.nlm.nih.gov/ under the accession no. MW645067. The associated BioProject, SRA, and BioSample numbers are PRJNA713943, SRR13948793, and SAMN18275439, respectively.
